# The role of closed-loop management of fever patients in a county-level medical community for the prevention and control of the post-coronavirus disease 2019 pandemic

**DOI:** 10.1097/MD.0000000000032690

**Published:** 2023-01-20

**Authors:** Yajun Ding, Chunxia Xu, Xingguo Zhang, Yanzhen He, Fang Wang, Libo Zhang, Yaner Yu, Yachun Zhou, Youping Zhang, Dongxian Ye

**Affiliations:** a Department of Prevention and Health Care, Beilun Branch of the First Affiliated Hospital, College of Medicine, Zhejiang University, Zhejiang, PR China.

**Keywords:** a county-level medical community, hierarchical medical system, post-coronavirus disease 2019 (COVID-2019) pandemic, the closed-loop management, the patients with fever, 2-way referral

## Abstract

We built a closed-loop management model for patients with fever in a county-level medical community and explored the role of this model in post-coronavirus disease 2019 (COVID-19) epidemic prevention and control. The subjects included 83,791 patients with fever treated in designated hospitals between February 2020 and April 2021. A pre-hospital, in-hospital, and post-hospital management system for patients with fever in the county-level medical community was established to allow the closed-loop management of these patients. SPSS software (version 13.0) was used to analyze the methods of visiting the hospital, nucleic acid detection in the hospital, and location of the patients after the hospital visit. Chi-square tests were used to compare the methods of visiting and location after hospital visits between patients with and without an epidemiological history. The number of patients with fever in the fever clinic showed a logarithm change (*R*^2^ = 0.4710), accompanied by seasonal changes. The number of fever patients with an epidemiological history decreased logarithmically monthly (*R*^2^ = 0.8876). Among patients with fever, 99.64% sought medical treatment on their own, with relatively low proportions undergoing home quarantine and requiring centralized quarantine special vehicles. After visiting the fever clinics, 98.56% of patients isolated at home or were monitored, with small proportions of patients requiring hospital admission or centralized isolation. However, the proportions of patients with home and centralized isolation with epidemiology were relatively high, accounting for 20.55% and 27.40% of cases, respectively. Compared to the overall population of patients with fever, the difference was statistically significant (*χ*^2^ = 48.881, *P* = .000). The establishment of a closed-loop management model for patients with fever in a county-level medical community strengthened the management of these patients. No local cases occurred in Beilun District between March 2020 and April 2021. In the post-COVID-19 era, all medical institutions in the county-level medical community strengthened infectious disease pre-examination and triage and promoted the formation of a strategic pattern of initial diagnosis at the grassroots level, 2-way referral, upper and lower linkage, and joint epidemic prevention. This management was more conducive to COVID prevention and control by hierarchical management according to the presence or absence of an epidemiological history.

## 1. Introduction

County-level medical communities focus on exploring the connection between integrated management and county-level hospitals as the leaders, township health centers as the hubs, and village clinics as the bases, forming a county-township-village 3-level medical care system and a 3-level linkage between county medical and health service systems. Between 2017 and 2019, >3000 county-level medical communities were established in China. The hierarchical diagnosis and treatment model of upper and lower (3-level) linkages was initially developed in domestic pilot counties.^[1,2]^

Zhejiang is one of 2 pilot provinces to promote the construction of compact medical communities in China.^[3]^ Since September 2017, Zhejiang has carried out a pilot study on this construction in 11 counties (cities, districts), which have integrated 39 county-level hospitals and 170 township medical institutions into 27 county-level medical communities.

Beilun District, the pilot medical community of Ningbo City, gathered the medical resources of the entire district to form 3 county-level medical communities. In February 2019, Beilun District People’s Hospital was the core hospital, which, combined with Daqi Street Community Health Service Center, Xiapu Street Community Health Service Center, Chunxiao Street Community Health Service Center, Meishan Street Community Health Service Center, and Binhai Hospital, comprised the Beilun District People’s Hospital Medical and Health Service Group, a county-based healthcare network.

In addition, our hospital is directly hosted by the First Hospital of Zhejiang University. Under the framework of the medical community, our hospital formed a 3-level province-county (district)-township medical service network. As a provincial-level county-level medical community pilot, our exploration and practice in medical resource sink provided an important reference for the “double sinking and two upgrade” strategy and the introduction of strong grassroots measures in Zhejiang Province to form model county-based healthcare networks in Beilun District.

In January 2020, the outbreak of the coronavirus disease 2019 (COVID-19) epidemic, county-level medical communities were tested by a sudden and major epidemic, in which the number of returnees increased sharply and people’s awareness and experience of epidemic prevention and the county’s epidemic prevention and anti-epidemic capabilities were weak.^[4]^ The primary medical and health institutions in the medical community of Beilun District implemented the homogenized management of patients with fever before the medical community, in, and after the hospital. Moreover, they identified factors that may have led to the spread of the epidemic and implemented interlocking prevention and control measures, achieved the closed-loop management of patients with fever, and cooperated with the local government to provide strong coordination and organization, resulting in a staged victory in epidemic prevention and control.

During the period of normalized epidemic prevention and control, small-scale epidemics still occurred in China. Ningbo-Zhoushan Port, the largest port worldwide in terms of throughput, is located in Beilun District. People frequently return from overseas, and the population in Beilun District exceeds 900,000 individuals. The pressure on epidemic prevention and control has been huge. The medical community of Beilun District People’s Hospital further standardized the closed-loop management of patients with fever at home and abroad; strengthened the prescreening, triage, and referral of suspicious patients; standardized nucleic acid testing and blood and imaging examinations; and implemented the classification and stratification of patients with fever. Post-hospital control provided a good model for the early detection of cases and patient isolation and treatment. As the only designated medical institution for fever clinics in the district, our hospital is also an institution for the treatment of suspected and confirmed cases of COVID-19, playing a critical role as a core hospital in the post-pneumonia epidemic period. This article describes the closed-loop management of patients with fever and its effects on prevention and control.

## 2. Materials and methods

This study was approved by the Ethics Committee of the College of Medicine, Zhejiang University. All procedures were performed in accordance with the ethical standards of the Declaration of Helsinki. Data were collected as part of public health surveillance and individual patient consent was obtained before collecting patient biological specimens. All identifiable patient-level data were anonymized.

### 2.1. Target populations and research methods

The research subjects were patients with fever visiting the fever clinic at Beilun People’s Hospital for treatment between February 2020 and April 2021. We analyzed the methods of presenting to the hospital, in-hospital examinations, and post-hospital management of these and carried out the entire process of their closed-loop management.

### 2.2. Closed-loop management of patients with fever

#### 2.2.1. Normalized pre-hospital management of patients with fever.

**Figure 1.**
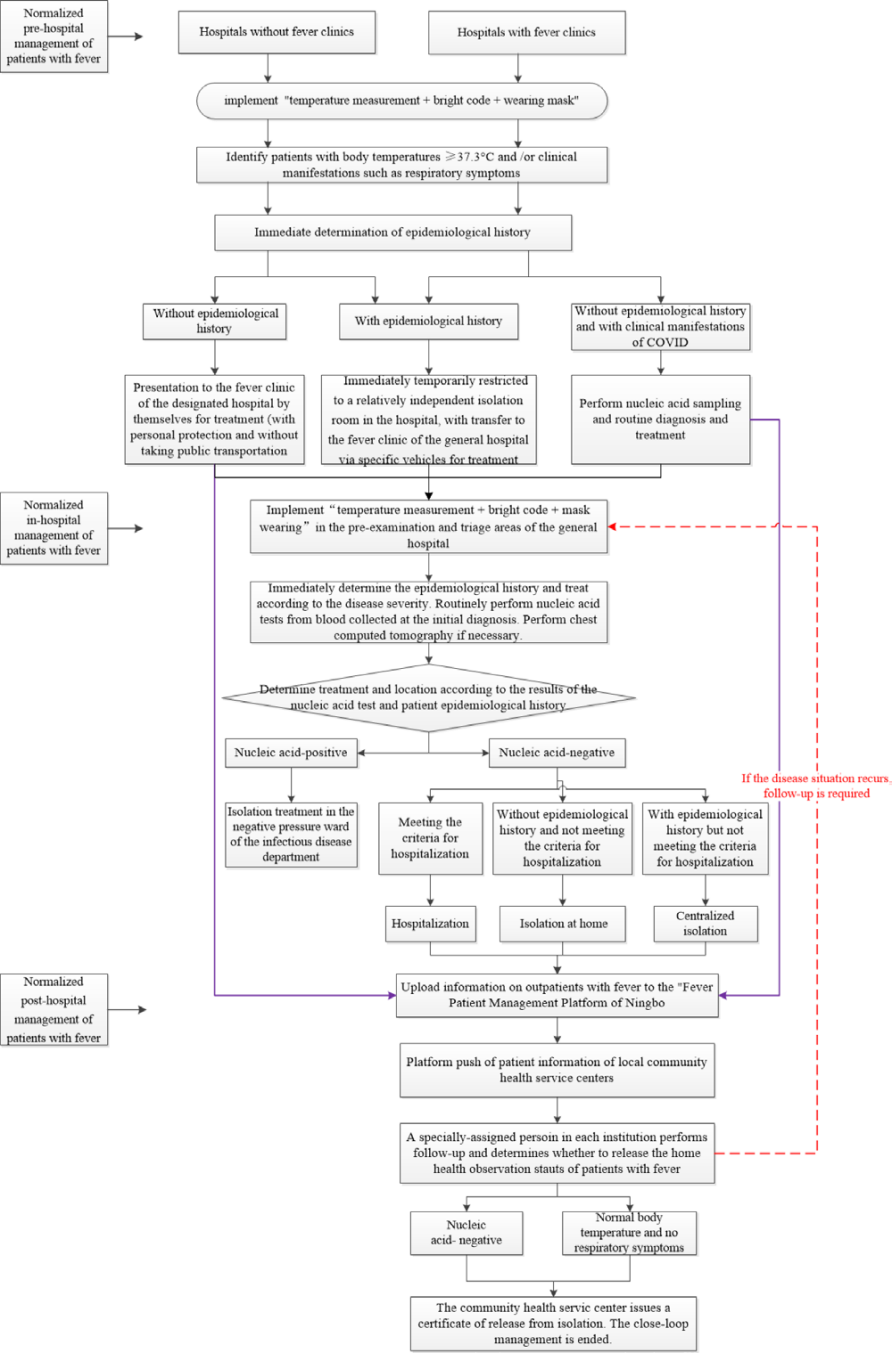
Flow chart of closed-loop management of patients with fever. The figure showed closed-loop management of the pre-hospital, in-hospital and post-hospital management of patients with fever. All institutions have strictly implemented the “temperature measurement + bright code + mask-wearing” system. All patients were required to pass the pre-examination to medical institutions to receive further medical treatment. Different treatments and locations were determined according to the results of the nucleic acid test and patients’ epidemiology. All fever outpatients’ information was uploaded to the primary medical and health institutions for follow-up management through the “Fever Patient’s Management Platform of Ningbo” after the treatment.

According to the “Administrative Measures for Pre-examination and Triage of Infectious Diseases in Medical Institutions”^[5]^ and the “New Coronary Virus Pneumonia Diagnosis and Treatment Plan (Trial Version 6),”^[6]^ county-based healthcare networks have formulated and continuously updated the prescreening processes in general hospitals and primary medical and health institutions (Fig 1). All institutions have strictly implemented the “temperature measurement + bright code + mask-wearing” system, such as patients and accompanying persons who were required to wear masks and maintain a distance of >1 meter; checked the personal “health code” information and determined the epidemiological history in detail. For special patients without a smartphone or unable to provide a health code, the first-diagnosis responsibility system was implemented, and manual inspection and registration were performed by verifying the ID cards and household registration books, and manually completing the flow adjustment form. All patients were required to pass the pre-examination to medical institutions to receive medical treatment.

#### 2.2.2. Normalized in-hospital management of patients with fever.

Hospitals without fever clinics were not permitted to receive patients with fever or respiratory symptoms. In patients with a body temperature ≥37.3°C detected by the infrared thermal imaging temperature measurement system or an abnormal health code, the basic information and epidemiological history were immediately determined, including the travel or residence history within 14 days before the disease onset; history of contact with other patients with fever or respiratory symptoms; engaging in high-risk occupations such as work related to imported cold chain food and goods; work in isolation areas, etc; history of clustered COVID-19 disease in life or workplace, especially travel history or residence history in key areas such as cities overseas or in China with medium and high-risk areas; previous confirmed history of COVID-19; history of contact with confirmed patients, etc.

Patients with fever without an epidemiological history who reported to the community health service center within 1 hour were directed to the fever clinic of the general hospital for treatment. Patients with negative nucleic acid test results after visiting the fever clinic of the general hospital were instructed to return to the primary health institutions for follow-up consultations. Patients with fever and an epidemiological history were immediately temporarily restricted to a relatively independent isolation room in the hospital district and the cases were reported to the community health service center. The community health service center called specific vehicles and transferred patients to the fever clinic of the general hospital for diagnosis and treatment.

In medical community hospitals with fever clinics, patients with a body temperature ≥37.3°C detected by the infrared thermal imaging temperature measurement system or an abnormal health code were directed by the previewing personnel to enter the temporary isolation room. Information on the epidemiological history and clinical manifestations including onset time and respiratory symptoms such as fever, cough, and other clinical symptoms was collected and the patients were classified and treated accordingly. Patients with no epidemiological history, clinical manifestations, or examination results not meeting the diagnostic criteria for COVID-19 underwent nucleic acid sampling and routine diagnosis and treatment in the fever clinic of the primary health institutions. Cases of patients with fever with epidemiological histories or in whom COVID-19 could not be ruled were immediately reported to the Medical Affairs of the District Health Bureau, special vehicles for transfer to the fever clinic of the general hospital were arranged, and patient referral registration and record handover were performed.

**Figure 2.**
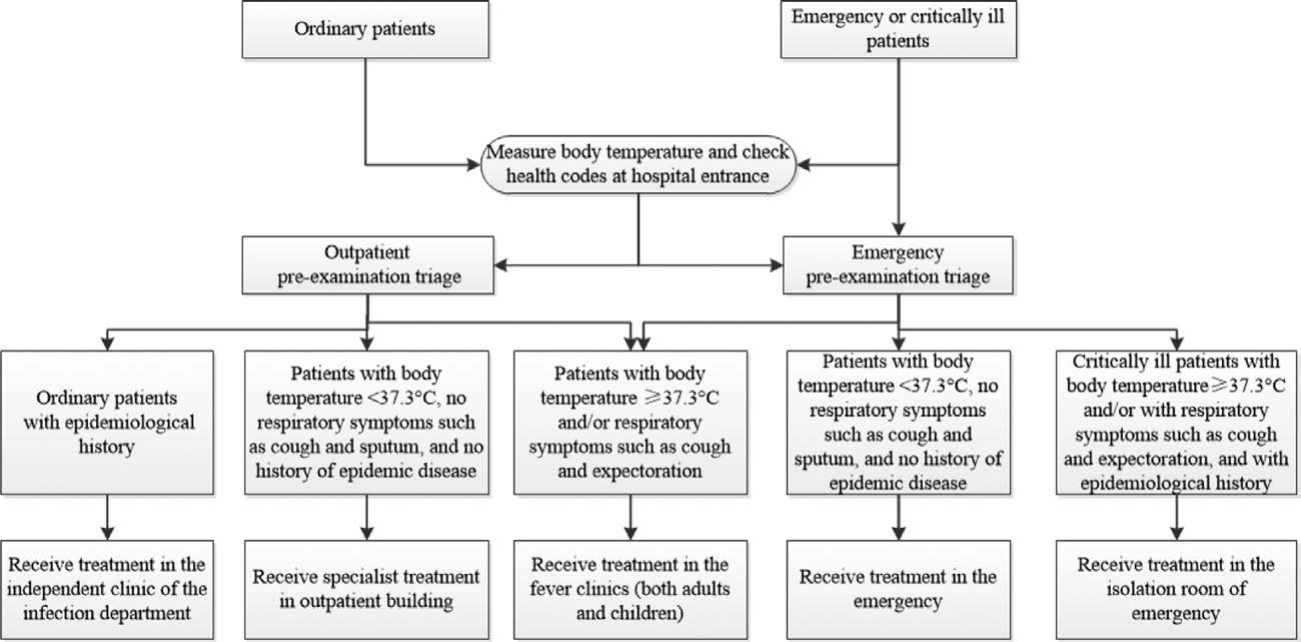
Flow chart of the closed-loop management of patients with fever in the designated general hospital. The figure showed that the general hospital with fever clinics set up pre-examination and triage offices at the entrance of the outpatient and emergency departments. The patients were shunted to different departments for treatment according to the body temperature, respiratory symptoms and epidemiology of fever patients.

The general hospital with fever clinics set up pre-examination and triage offices at the entrance of the outpatient and emergency departments. Patients with a body temperature ≥37.3°C detected by the infrared thermal imaging temperature measurement system or an abnormal health code were accompanied by pre-inspection personnel to the fever clinic throughout the entire process. General hospitals standardized the construction of independent quarantine areas in the emergency department for patients with acute and severe fever or epidemiological history (Fig 2).

**Figure 3.**
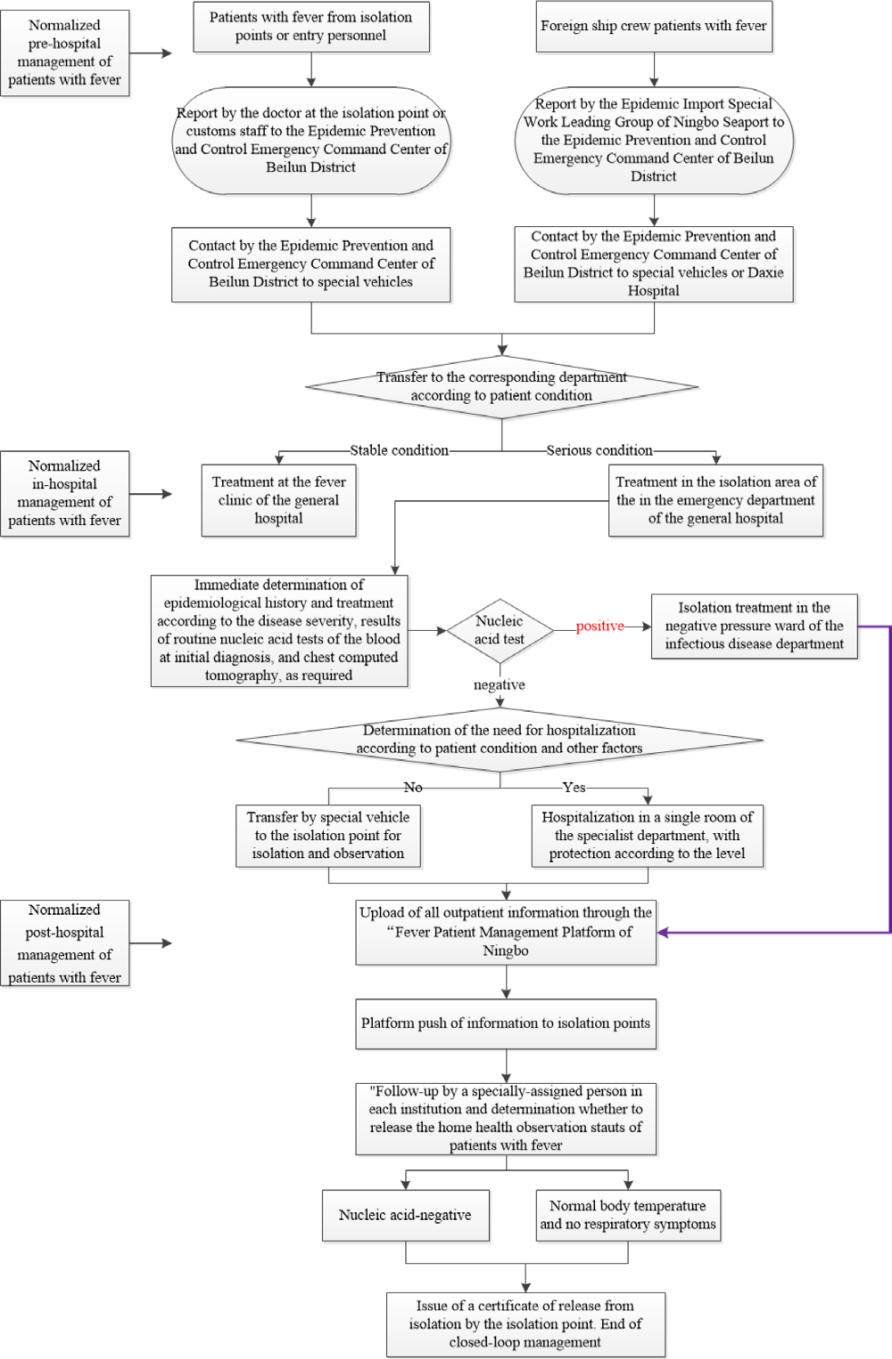
Flow chart of the closed-loop management of patients with fever at isolation points and among entry personnel. The figure showed the closed-loop management of the pre-hospital, in-hospital and post-hospital management of patients with fever at isolation points and among entry personnel. Different from common fever patients, the patients with fever at isolation points and among entry personnel were transferred by special vehicle to the fever clinic of the general hospital for treatment though the Ningbo Seaport Special Work Leading Group for Epidemic Importation notified the Beilun District Emergency Command Center. Different treatments and locations were determined according to the results of the nucleic acid test and disease severity. All fever patients’ information was uploaded to the primary medical and health institutions for follow-up management through the “Fever Patient’s Management Platform of Ningbo” after the treatment.

After the first negative round of new COVID-19 nucleic acid test results at customs, patients with fever who were crew of foreign ships or who were travelers, the Ningbo Seaport Special Work Leading Group for Epidemic Importation notified the Beilun District Emergency Command Center, which then contacted Beilun District 120 First Aid Center or Daxie Hospital. The patients were then transferred by special vehicle to the fever clinic of the general hospital for treatment. Crew members with acute and severe fever were treated in the independent quarantine areas of the emergency department (Fig 3).

The fever clinic of the general hospital standardized the examination of patients with fever. Nurses in the triage area checked and confirmed patients with fever during the initial preview. All patients were treated according to the disease severity and routinely tested for viral nucleic acid in the blood at the initial diagnosis. Chest computed tomography was also performed. Reexamination patients underwent repeated nucleic acid detection tests, depending on the situation. The nucleic acid test results of patients in the fever clinic were reported within 4 to 6 hours. The patients were instructed not to move around at will and they could only leave the fever clinic after achieving nucleic acid test results.

#### 2.2.3. Normalized post-hospital management of patients with fever.

All fever outpatient information was uploaded to the primary medical and health institutions for follow-up management through the “Fever Patient’s Management Platform of Ningbo.” Specially assigned persons in each institution were responsible for follow-up and determining whether to release the home health observation status of patients with fever. Tracking was terminated in patients with fever with negative nucleic acid test results. In patients with fever who did not undergo nucleic acid tests, closed-loop management was terminated only after their body temperature returned to normal and no respiratory symptoms were present. Persons at the isolation point, patients who were crew of foreign ships, and travelers were transported by special vehicles to the isolation point for continued isolation and observation. Fever patient and follow-up management requirements were adjusted promptly for COVID-19 epidemic control.

### 2.3. Statistical analysis

All clinical data of the 83,791 patients with fever between February 2020 and April 2021 were provided by the fever clinic of the General Hospital. Statistical analyses of hospital visits, nucleic acid detection, and locations of patients with fever were conducted using SPSS version 13.0 (SPSS Inc., Chicago, IL). Chi-squared tests were used to compare the methods of presenting to the hospital and the whereabouts of fever patients with and without epidemiological histories. The multiple linear regression analysis was adopted to calculate partial correlation coefficient of the numbers of fever patients and the fever patients with epidemic history. The multiple linear regression analysis was adopted to calculate partial correlation coefficient of the numbers of fever patients and the fever patients with epidemic history. Statistical significance was set to *P* < .05.

## 3. Results

### 3.1. Numbers and trends of patients with fever

**Figure 4.**
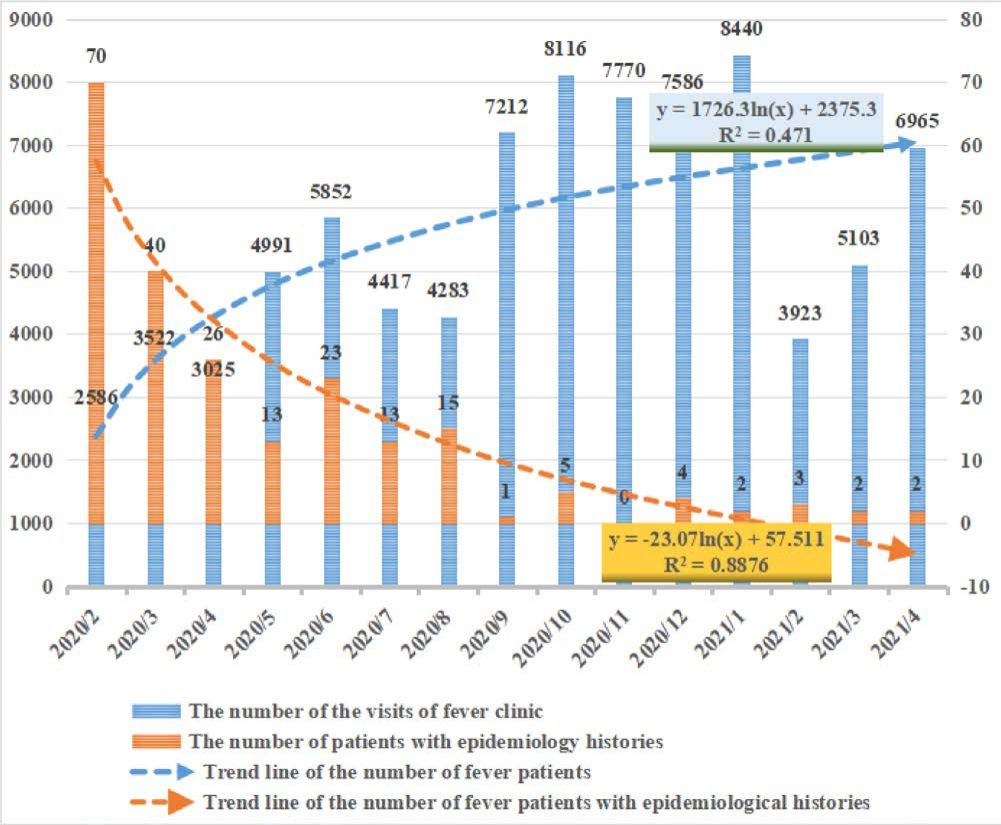
Visit numbers and trends of patients at fever clinics between February 2020 and April 2021. The figure showed that the number of patients with fever in the fever clinic showed a logarithm change (*R*^2^ = 0.4710), accompanied by seasonal changes. The number of fever patients with an epidemiological history decreased logarithmically monthly (*R*^2^ = 0.8876).

Since the outbreak of COVID-19, from February 2020 to April 2021, the fever clinics (including adult and child fever clinics) of designated hospitals received and managed 83,791 patients with fever. The number of fever clinic visits gradually increased from February 2020 to January 2021 (in 1 year) and decreased in February of the following year. The multiple linear regression analysis showed a logarithmic change in the number of patients with fever between February 2020 and January 2021 (y = 1726.3ln(x) + 2375.3, R2 = 0.4710). Meanwhile, the number of fever patients increased monthly from February to June, dropped slightly from July to August, then increased significantly, dropped sharply in February of the following year, and then continued to rise again. The number of patients with fever in fever clinics between February and April 2021 increased compared to the same period in 2020 (Table 1 and Fig 4). The 219 patients with fever with an epidemiological history accounted for 0.26% of all patients with fever. The number of patients with fever with an epidemiological history was highest in the early stage of the COVID-19 epidemic, then decreased monthly. The multiple linear regression analysis showed a logarithmic decrease in the number of patients with fever with an epidemiological history (y = −23.07ln(x) + 57.511, R2 = 0.8876) (Table 2, Fig 4).

**Table 1:** Composition ratios of the methods for presenting to the hospital and the nucleic acid detection rates of patients with fever. N (%).

Yr/Mo	Number of visits to the fever clinic	Methods of presentation to the hospital			Nucleic acid detection rates of patients with fever
		Ordinary fever patients seeking medical care on their own	Fever patients at home seeking medication by special vehicles	Fever patients in the centralized isolation area seeking medication by special vehicles	
2020/2	2586	2514 (97.22)	33 (1.28)	39 (1.51)	2586 (100.00)
2020/3	3522	3470 (98.52)	18 (0.51)	34 (0.97)	3522 (100.00)
2020/4	3025	3002 (99.24)	0 (0.00)	23 (0.76)	3025 (100.00)
2020/5	4991	4985 (99.88)	0 (0.00)	6 (0.12)	4991 (100.00)
2020/6	5852	5808 (99.25)	43 (0.73)	1 (0.02)	5852 (100.00)
2020/7	4417	4397 (99.55)	18 (0.41)	2 (0.05)	4417 (100.00)
2020/8	4283	4255 (99.35)	25 (0.58)	3 (0.07)	4283 (100.00)
2020/9	7212	7189 (99.68)	18 (0.25)	5 (0.07)	7212 (100.00)
2020/10	8116	8107 (99.89)	5 (0.06)	4 (0.05)	8116 (100.00)
2020/11	7770	7769 (99.99)	1 (0.01)	0 (0.00)	7770 (100.00)
2020/12	7586	7584 (99.97)	0 (0.00)	2 (0.03)	7586 (100.00)
2021/1	8440	8427 (99.85)	9 (0.11)	4 (0.05)	8440 (100.00)
2021/2	3923	3919 (99.90)	0 (0.00)	4 (0.10)	3923 (100.00)
2021/3	5103	5100 (99.94)	0 (0.00)	3 (0.06)	5103 (100.00)
2021/4	6965	6965 (100.00)	0 (0.00)	0 (0.00)	6965 (100.00)
Total	83791	83491 (99.64)	170 (0.20)	130 (0.16)	83791 (100.00)

**Table 2:** Composition ratios of the locations of patients with fever. N (%).

Yr/Mo	Number of the visits to the fever clinic	Composition ratios of the locations of patients with fever			Patients with epidemiology histories	Composition ratios of the locations of patients with fever with epidemiology histories			
		Patients isolated or monitored at home	Hospitalized patients	Patients isolated in centralized isolation points		Patients isolated or monitored at home	Patients hospitalized in the general hospital	Patients isolated in centralized isolation points	Patients hospitalized in Binhai hospital
2020/2	2586	2488 (96.21)	62 (2.40)	36 (1.39)	70 (2.71)	44 (62.86)	5 (7.14)	14 (20.00)	7 (10.00)
2020/3	3522	3404 (96.65)	89 (2.53)	29 (0.82)	40 (1.14)	17 (42.50)	9 (22.50)	14 (35.00)	0 (0.00)
2020/4	3025	2936 (97.06)	74 (2.45)	15 (0.50)	26 (0.86)	7 (26.92)	13 (50.00)	6 (23.08)	0 (0.00)
2020/5	4991	4961 (99.40)	26 (0.52)	4 (0.08)	13 (0.26)	8 (61.54)	2 (15.38)	3 (23.08)	0 (0.00)
2020/6	5852	5816 (99.38)	35 (0.60)	1 (0.02)	23 (0.39)	13 (56.52)	9 (39.13)	1 (4.35)	0 (0.00)
2020/7	4417	4393 (99.46)	21 (0.48)	3 (0.07)	13 (0.29)	10 (76.92)	0 (0.00)	3 (23.08)	0 (0.00)
2020/8	4283	4228 (98.72)	54 (1.26)	1 (0.02)	15 (0.35)	12 (80.00)	2 (13.33)	1 (6.67)	0 (0.00)
2020/9	7212	7139 (98.99)	73 (1.01)	0 (0.00)	1 (0.01)	0 (0.00)	1 (100.00)	0 (0.00)	0 (0.00)
2020/10	8116	7998 (98.55)	114 (1.40)	4 (0.05)	5 (0.06)	3 (60.00)	2 (40.00)	0 (0.00)	0 (0.00)
2020/11	7770	7676 (98.79)	94 (1.21)	0 (0.00)	0 (0.00)	0 (/)	0 (/)	0 (/)	0 (/)
2020/12	7586	7470 (98.47)	116 (1.53)	0 (0.00)	4 (0.05)	0 (0.00)	4 (100.00)	0 (0.00)	0 (0.00)
2021/1	8440	8305 (98.40)	135 (1.60)	0 (0.00)	2 (0.02)	0 (0.00)	1 (50.00)	1 (50.00)	0 (0.00)
2021/2	3923	3878 (98.85)	44 (1.12)	1 (0.03)	3 (0.08)	0 (0.00)	2 (66.67)	1 (33.33)	0 (0.00)
2021/3	5103	5030 (98.57)	73 (1.43)	0 (0.00)	2 (0.04)	0 (0.00)	1 (50.00)	1 (50.00)	0 (0.00)
2021/4	6965	6861 (98.51)	104 (1.49)	0 (0.00)	2 (0.03)	0 (0.00)	2 (100.00)	0 (0.00)	0 (0.00)
Total	83791	82583 (98.56)	1114 (1.33)	94 (0.11)	219 (0.26)*	114 (52.05)	53 (24.20)	45 (20.55)	7 (3.20)

* Statistically significant difference in the location of patients with fever with epidemiological histories compared to the whole group of patients with fever (*χ*^2^ = 48.881, *P* = .000).

### 3.2. Methods of visiting the hospital, nucleic acid detection, and the locations of patients with fever

Among the 83,791 patients with fever, 83,491 (99.64 %) sought medical treatment by themselves. A total of 170 patients in home isolation were sent to the fever clinic by special car, accounting for 0.20% of cases; 130 (0.16%) of patients in the centralized isolation area were sent to a fever clinic by special car. When visiting the fever clinic, the nucleic acid detection rate of the fever clinic was 100.00%. After seeing a doctor, 98.56% of patients were isolated or monitored at home, 1.33% required hospitalization due to illness, and 0.11% were transported by special vehicles to a centralized isolation point for isolation. The locations of patients with fever were generally consistent with the methods of visiting the hospital. In this study, 219 patients with fever had an epidemiological history, accounting for 0.26% of all patients with fever. After treatment, 52.05% of patients underwent isolation at home, 20.55% isolated in a centralized isolation area, and 27.40% were hospitalized. The difference in the locations of fever patients with epidemiological histories compared to the whole group of patients with fever patients was statistically significant (*χ*^2^ = 48.881, *P* = .000) (Table 2).

## 4. Discussion

Between February 2020 and April 2021, fever clinics (including adult and child fever clinics) treated 83,791 patients with fever. The number of visits to fever clinics showed a parabolic change, increasing gradually from February 2020 to January 2021 and decreasing in February 2021. Owing to the Chinese Festival in February, migrants returned to their homes; thus, the number of fever visits dropped sharply. After the Chinese Festival, migrants returned to Beilun District, and the number of fever patients increased rapidly, demonstrating the effects of social factors. The number of patients with fever also decreased during hot months (July–August), showing the seasonal effects, with increased cases in winter and spring and decreased cases in summer months.

Strengthening the screening and closed-loop management of patients with fever is important to stay ahead of the virus and the detection of COVID-19. Closed-loop management is a modern hospital management system comprising integrated management control, information systems, closed-loop systems, and other principles. During the COVID-19 outbreak, all medical institutions in the county-level medical community set the unification target and standardized process for preview triage. The preview triage personnel consisted of the medical staff, who improved the preview triage accuracy and effectiveness,^[7]^ and ensured close connections to the primary health institutions. Patients with fever and a history of epidemiology or suspected patients, including those from concentration isolated areas and foreign workers were docked point-to-point and treated promptly. The up-down linkage and 2-way referral between the general hospital and primary health institutions effectively ensured that patients with fever were transferred to designated fever clinics for treatment to reduce the risk of transmission and effectively improve epidemic prevention and control in county-based healthcare networks.^[8,9]^

Each medical institution strengthened the in-hospital management of fever patients, standardized the medical treatment procedures for patients with fever as required, and established the "four lines of defense" called the "closed-loop management", which included preview in general and emergency outpatients, detailed consultation in the general and emergency departments, screening in fever clinics, and guard a pass by an expert team.

Closed-loop management in this study ensured quick diagnosis, centralized treatment, strict management, and effective shunting of patients with fever after entering the fever clinic. All newly diagnosed fever patients were required to undergo nucleic acid testing to rule out COVID-19 infection. Simultaneously, the county-level medical community made full use of the initially established remote diagnosis and treatment platform. The general hospital conducted timely online consultations with designated provincial hospitals based on a remote consultation platform. The online system of epidemic treatment to provide a remote connection between provincial designated hospitals and grassroots medical institutions relieved pressure on hospitals to provide medical treatment, reduced the opportunity for people to gather, and reduced the risk of cross-infection.^[10]^

The post-hospital management of patients with fever is mainly based on the corresponding treatment of patients from different sources, which is crucial for the prevention and control of the COVID-19 epidemic and is also a key part of closed-loop management. The primary health institutions were embedded in community grid management. Post-hospital management, the follow-up of patients with fever after treatment, was carried out by specially-assigned persons from the primary health institutions according to the “Fever Patient’s Management Platform of Ningbo.” These persons strengthened the temperature measurement, investigation, and monitoring of key sentinel points; conducted follow-up investigations; and supervised home isolation or monitored personnel.

In this study, based on patient condition and source, the fever clinic of the general hospital transferred patients to the isolation point for isolation, to the home for health management, or for treatment in the hospital. The proportions of patients with fever were consistent with the patient source. Patients with fever with epidemiological histories were more likely to be transferred to centralized isolation points or hospitalized for isolation than ordinary fever patients, which is more conducive to high-risk patients receiving nucleic acid testing more quickly and to patients with COVID-19 receiving treatment in the hospital. At the same time, strengthening control of risk groups and avoiding contact with others can reduce the risk of community transmission.

The implementation of closed-loop management of fever patients also requires certain conditions. For example, the number of patients needs to match the scope of treatment of the designated medical institution. If the fever patients have a blowout and hospital systems become quickly overwhelmed, ambulatory strategies need to be adjusted. A study in New York showed that when the coronavirus disease (COVID-19) pandemic swept New York City in 2019, they designed and implemented an innovative program called the Cough Cold and Fever Clinic. Patients with symptoms suspicious for COVID-19 were first triaged via telemedicine to determine necessity of in-person evaluation, and then conducted treatment and follow-up. The overall mortality rate during the pandemic peak has been greatly reduced through telemedicine.^[11]^ This is an experience worth learning. In addition, if COVID-19 patients conceal their fever and do not seek medical treatment in time, it will also lead to the spread of the epidemic. Therefore, to implement such a closed-loop management requires not only the full cooperation of all government departments for the formulation and implementation of an unified management system and process, but also the solid economic support from country (because fever patients are treated mostly free of charge). The most important is the social mobilization to improve the participation of the public, establish the concept of “everyone is the first responsible person for health,” and actively cooperate with the national epidemic prevention policy. Such closed-loop management only could be completed by country-society-public tripartite linkage, which has played a very good role in curbing the spread of the virus at the beginning of the COVID-19 pandemic and was also an important exploration and successful practice of prevention and control in COVID-19 in China.

## 5. Conclusion

No local cases occurred in the jurisdiction between March 2020 and April 2021 through the closed-loop management model of patients with fever in the county-level medical community. Thus, in the period following the COVID-19 pandemic, it is crucial to form 3-level “guarantee” networks and establish a joint prevention and control mechanism for county, township, and village medical institutions^[10,12,13]^ to more effectively provide epidemic prevention and control. General hospitals should devote major resources to the diagnosis and treatment of acute and critical patients and play a role in technical radiation and guidance for primary institutions. With the support of the leading general hospital, the primary institutions should fully perform their outpost role in epidemic prevention and control, while strengthening the post-hospital management of patients with fever to ensure that closed-loop management is a strategic pattern of 2-way referral, upper and lower linkage, and joint epidemic prevention.

## Author contributions

**Conceptualization:** Chunxia Xu, Xingguo Zhang, Yanzhen He.

**Data curation:** Yanzhen He, Fang Wang, Dongxian Ye.

**Formal analysis:** Fang Wang.

**Investigation:** Yanzhen He, Fang Wang, Dongxian Ye.

**Methodology:** Libo Zhang, Dongxian Ye.

**Project administration:** Yajun Ding, Libo Zhang, Yaner Yu.

**Resources:** Libo Zhang, Yaner Yu.

**Supervision:** Yajun Ding, Chunxia Xu, Xingguo Zhang, Yachun Zhou, Youping Zhang.

**Validation:** Yajun Ding, Chunxia Xu, Xingguo Zhang, Yachun Zhou, Youping Zhang.

**Visualization:** Yachun Zhou.

**Writing – original draft:** Yaner Yu, Dongxian Ye.

**Writing – review & editing:** Dongxian Ye.
